# Visibility of alveolar bone thicknesses on CBCT images–a study on minimum bone requirements using various reconstruction techniques, viewing modes, and resolutions

**DOI:** 10.1007/s00784-024-06034-1

**Published:** 2024-11-15

**Authors:** Camilla Lennholm, Hanna Andreasen, Anna Westerlund, Henrik Lund

**Affiliations:** 1 Department of Oral and Maxillofacial Radiology, Institute of Odontology, Sahlgrenska Academy, Gothenburg University, PO Box 450, Gothenburg, SE-405 30 Sweden; 2 Oral and Maxillofacial Radiology Department, Region Hospital Halland, Halmstad, Sweden; 3 Department of Orthodontics, Institute of Odontology, Sahlgrenska Academy, Gothenburg University, Gothenburg, Sweden

**Keywords:** CBCT, Cone-beam computed tomography, Histology, Orthodontics, Periodontology, Radiology

## Abstract

**Objectives:**

To evaluate at which thickness marginal bone becomes visible to the observer on cone-beam computed tomography (CBCT) images and how reconstruction technique and viewing mode affect assessment.

**Materials and methods:**

Fourteen anterior teeth from six human mandibles were examined with two CBCT resolution protocols: standard- and high-resolution. Distance from the cementoenamel junction to the visible marginal bone level (MBL) was measured in three groups of reconstructed CBCT images: multiplanar reformation (MPR) with grey scale, MPR with inverted grey scale, and 3D rendering. These measurements were used to identify the bone level where marginal bone width should be measured on histological photographs of sliced teeth. Gold standards comprised measurements of bone thickness at the superior MBL on histological photographs.

**Results:**

MPR grey scale images exposed at high-resolution settings yielded highest validity: bone widths of 0.173 mm (buccal) and 0.356 mm (lingual) were necessary for visibility on a CBCT image. 3D-rendered lingual surfaces exposed with high-resolution settings had lowest validity. Intra-observer agreement for all CBCT and histological measurements was high.

**Conclusion:**

The best CBCT resolution protocol, reconstruction technique, and viewing mode for analyzing buccal and lingual surfaces of the alveolar bone margin are images exposed with a high-resolution protocol, reconstructed using MPR, and viewed in grey scale. Bone thickness required to be visualized was twice lingually compared to buccally.

**Clinical relevance:**

The visualization of bone thickness in CBCT requires a greater thickness on the lingual side compared to the buccal side. 3D-rendered reconstructions should be avoided when evaluating thin bony structures.

## Introduction

Since its introduction, cone-beam computed tomography (CBCT) has been used in diagnosis and treatment planning in several dental disciplines for tasks, such as tooth localization before extraction, pre-surgical investigation of pathological conditions, and evaluation of the sinus floor before dental implant installation [1, 2]. The technique is also frequently used in orthodontic research on canines and for evaluating the negative effects of orthodontic treatment such as root resorption and decreasing bone level [3]. With this technique, we have, for example, shown that some orthodontic patients are more prone to experiencing a lowered bone level during orthodontic treatment, particularly those who have a pretreatment basal open vertical relationship, posterior rotation of the mandible, or incisor protrusion [4].

A CBCT image is a 3-dimensional (3D) reconstruction where stacks of 2-dimensional (2D) images in each orthogonal plane (axial, coronal, and sagittal) can be scrolled through and explored. These images should therefore be displayed and interpreted digitally as the viewer can make adjustments to realign the data set to fit the patient’s anatomic features. Various options are available for displaying the data. Many software programs can reformat the data in a way that highlights the desired component of the volumetric set. The two basic options are multiplanar reformation (MPR) and volumetric (3D) rendering. Conversion to MPR creates non-axial, 2D images and enables the volumetric dataset to be viewed in other non-orthogonal planes better suited for maxillofacial structures, such as linear oblique and curved planes. Volumetric rendering makes possible visualization of voxels within a selective data set and uses either an indirect or a direct technique. Indirect volumetric rendering reconstructs volumetric surfaces with depth. Direct volumetric rendering creates a volumetric data set comprising voxel grey intensities within a specified threshold and eliminates values below or above the threshold [5].

There are well known technical limits to spatial resolution in a CBCT-image, e.g. the capability of displaying fine detail. These relate both to voxel size as well as to generate information theory and other factors such as image noise [6–8].

Earlier studies have had the aim to measure the accuracy of bone height and width displayed in CBCT reconstructions and to compare how this changes when the CBCT settings are adjusted in the image-acquiring stage. Gerlach et al. [9] concluded that CBCT imagery tends to overestimate the anatomical truth of bony structures, both in width and height, and especially in cortical bone.

Thönissen et al. found that thin bony structures of a thickness less than 0.5 mm could not be reliably depicted in CBCT images [10]. However, Patcas et al. [11] concluded that, using a high-resolution protocol with a voxel size of 0.125 mm, an alveolar bone thickness of even 1 mm could be misinterpreted.

The recent Lennholm et al. [12] study found that thin bony structures were not reliably visible on CBCT images compared with histological measurements; CBCT measurements systematically exceeded histological measurements. These results raised the issue of the minimal bone widths required in order for bone thicknesses to be visualized on CBCT images. Moreover, with the emergence of new techniques in orthodontics, such as aligners, fewer extractions are being performed as crowding is increasingly being resolved through expansion. Expansion, however, might lead to the positioning of the teeth outside the bone envelope, potentially causing future recessions. For proper assessments to be made when using CBCT for treatment planning and follow-up, the lower limit of how much bone this technique can reliably depict must be understood.

Thus, the aim of the study was to evaluate at which bone width the marginal bone level becomes reliably visible on CBCT images and how different reconstruction techniques and viewing modes influence visibility.

## Materials and methods

### Material

The material comprised 14 anterior mandibular teeth (incisors and canines) from six human specimens. The same material used in CBCT imaging was used in construction of the histologic gold standard. The Ethics Review Committee at Uppsala approved the study protocol (Daybook no. [Dnr]: 2019 − 01582).

Each tooth received an identification code, in addition to a metal ligature that was attached along the long axis before the radiographic examination. Because the CBCT examinations were done before the teeth were sliced, the ligature functioned as a marker for positioning during CBCT measurements in relation to the histologic Sect. [12].

### CBCT examination and measurement procedure

Each mandible was surrounded by a soft tissue-imitating material before examination (Superflab, Eckert & Ziegler, BEBIG GmbH, Berlin, Germany) and imaged with the Accuitomo 170 3D CBCT unit (J. Morita Mfg. Corp, Kyoto, Japan). A full 360-degree rotation was made during imaging, and exposure parameters were set to 75 kV; 5 mA; field of view, 60 ✕ 60 mm; and voxel size, 0.125 mm. Each tooth was imaged once at standard resolution (STD) and once at high resolution (HI). The image data, contiguous axial slices, were exported in the Digital Imaging and Communications in Medicine (DICOM) format with a slice thickness of 0.5 mm (121 images) for the standard-resolution and 0.125 mm (481 images) for the high-resolution protocols; they were later imported, visualized and analyzed in OstriX MD (Pixmeo SARL, Bernex, Switzerland) [12]. Parameters such as FOV, kV and mA were kept constant at exposure in order to discern the influence of technical properties and exposure parameters on CBCT visualization. Differences in slice thickness and interval of exported base images were made to compare the day-to-day clinical setup with a proposed optimal setup.

**Fig. 1:**
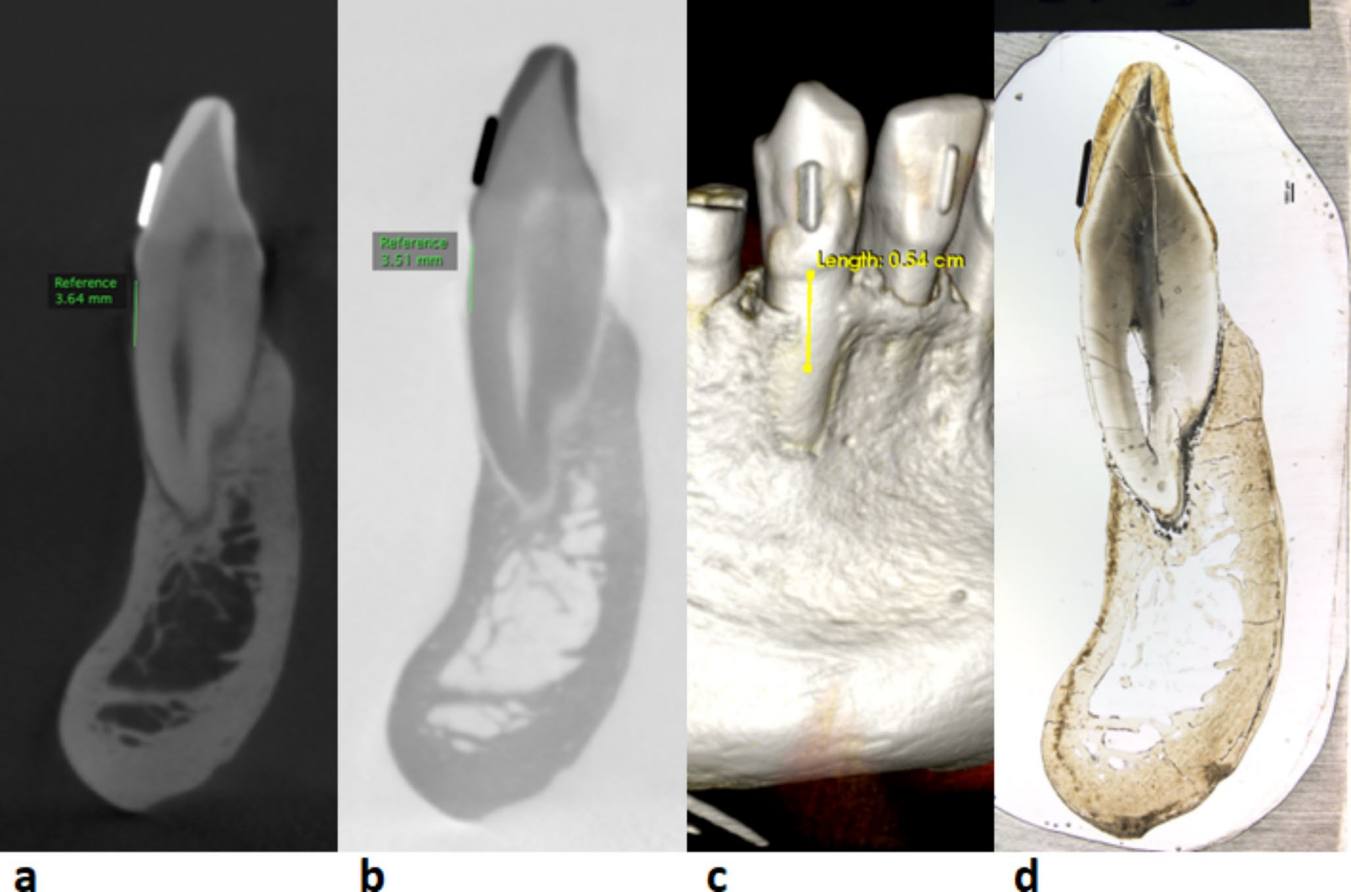
Cone-beam computed tomography reconstructions and the histological image of a mandibular canine. **A** Multiplanar reformation (MPR) and the grey scale viewing mode. **B** MPR and the inverted grey scale viewing mode. **C** 3D rendering. **D** Photograph of the corresponding histological cut

The imaging data was prepared for analysis using multiplanar reformation (MPR) by orienting the image planes in axial, coronal and sagittal views related to the long axis of the tooth and the ligature. During the reformation process, CBCT images were aligned using the photographed histological section as a template to ensure a correct position of the CBCT image plane in relation to the histological section. The reformations enabled optimal visualization of the tooth and surrounding marginal bone level in three images planes. A single CBCT image in the sagittal view, showing the buccal and lingual bone corresponding to the photographed histological section, was saved for later analysis and measurement. A specialist in dentomaxillofacial radiology (HL) made the following measurements. On the CBCT images, measurements were made from the cementoenamel junction (CEJ) to the marginal bone level (MBL; Fig. 1). For each tooth, the buccal and lingual sites were measured using two reconstruction formats: (1) 3D-rendering and (2) MPR in two viewing modes: grey scale and inverted grey scale. Measurements in these three viewing groups (3D-rendering, MPR grey scale, and MPR inverted grey scale) were made twice for each tooth: on standard- and on high-resolution images. The 3D-rendered images were measured after the image had been optimized for bone visualization. On each CBCT image, the CEJ-MBL distance was measured twice and averaged. This distance would later serve to indicate where to measure bone width on the histological photograph.

### Histological preparation and measurement procedure

Following the CBCT examinations, we prepared the 14 tooth specimens as described by Donath (1988) [13]: they were cut along the long axis, ground to a thickness of 100 μm, and photographed using a digital camera (Nikon DS-Fi1) through a light microscope (Nikon SMZ 800; Tokyo, Japan, 0.5X lens (X 1–6.4) with a magnification of 10 × (0.5 × 1 × 10). Nikon documentation software was used. Images were saved as.tif files, and a calibration marker of 1000 μm was visible in each image. The 14 photographs were viewed and measured in ImageJ, an image processing program (Image J2, Version 2.3.0/1.53q, Build: d544a3f481, Date: 2021-09-13, Open-source image processing software, Copyright 2010–2023), using the 1000-µm calibration marker for calibrating scale before taking measurements.

**Fig. 2:**
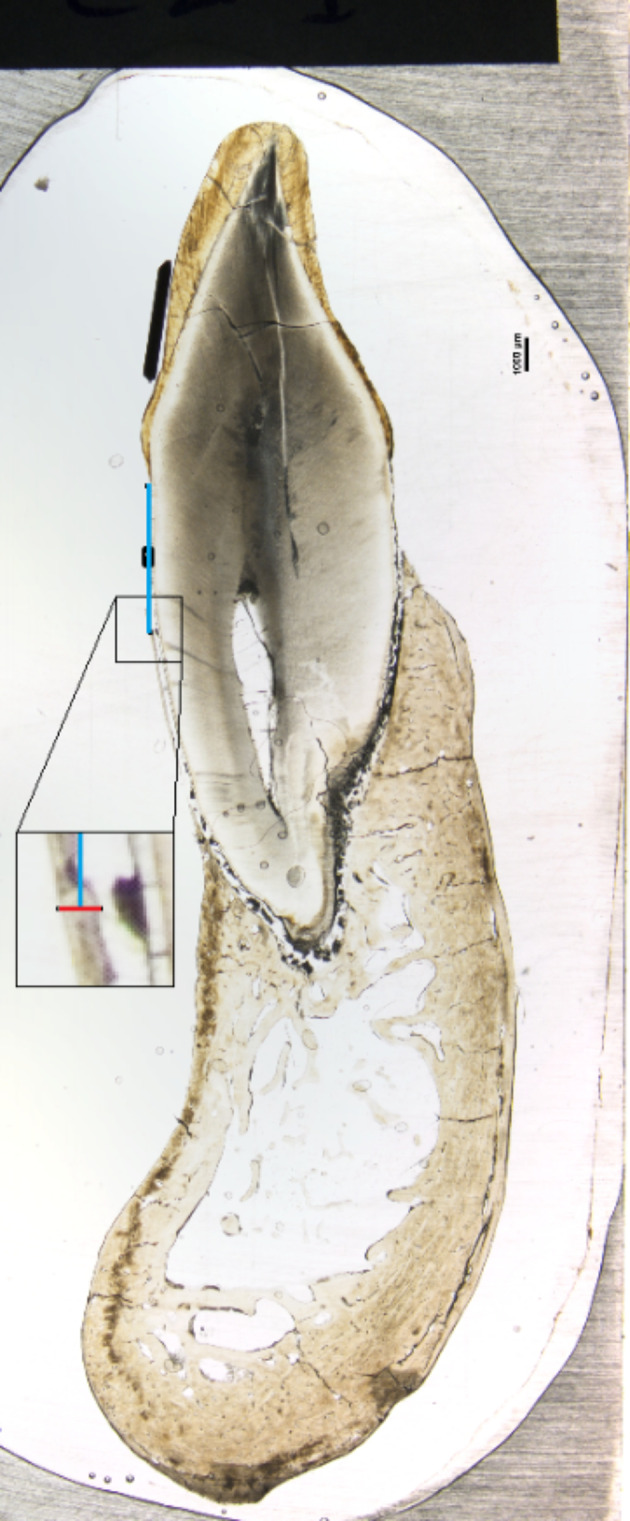
Simplified model of a histological measurement on a photograph of a histological cut tooth (here, the buccal measurement). Blue vertical line: CBCT reference measurement from the cementoenamel junction to the visible bone margin. Red horizontal line: bone width recorded at the level of the CEJ-MBL reference distance

Measurement sites of the buccal and lingual aspects were marked on the photographs of the histological preparations (Fig. 2) using the reference distance (CEJ-MBL) obtained from the three CBCT image-viewing groups, on both standard- and high-resolution images. Thereafter, one observer (HA) assessed bone width twice for the buccal and twice for the lingual aspects. To determine the gold standards (one buccal and one lingual gold standard for each tooth), another observer (HL) made five marginal bone thickness measurements on the buccal and five on the lingual surfaces. Measurements were made at the superior, visible position, that is, the thinnest part of the marginal bone.

All the measurements in the present study were performed with an interval of at least 14 days.

### Statistical analyses

Intra-observer agreement for all histological and CBCT measurements was assessed using the intraclass correlation coefficient (ICC).

Descriptive statistics and boxplots were used to compare the viewing groups with their gold standards. The Wilcoxon signed-rank test was used to compare paired samples. The significance level was set at *p* < 0.05. The Statistical Package for the Social Sciences (IBM^®^ SPSS^®^, version 27.0; IBM Inc., Chicago, Illinois, USA) or GraphPad Prism (version 8.3.1; GraphPad Software, San Diego, California, USA) was used for all statistical analyses.

## Results

### Reliability

Intra-observer agreement for measurements on the CBCT images had ICC ranges of 0.91–0.99 for the standard-resolution protocol and 0.93–0.98 for the high-resolution protocol. For histological measurements, based on reference values from CBCT, intra-observer agreement had ICC ranges of 0.94–1.00 for the standard-resolution protocol and 0.87–1.00 for the high-resolution protocol.

Of the 84 surfaces (*n* = 42 buccal and 42 lingual; all three viewing groups) depicted on the standard-resolution CBCT images, marginal bone level of 18 surfaces (21.4%) had been measured at a height that, on the histologic images, included no visible bone. The 18 surfaces comprised the following sites: eight buccal and two lingual (MPR grey scale), five buccal (MPR inverted grey scale), and three buccal and two lingual (3D rendered). The same phenomenon occurred on the CBCT high-resolution images: on 16 of the 84 surfaces (19.0%); marginal bone level had been measured at a height that, in the histology images, included no visible bone. The 16 surfaces comprised the following sites: three buccal and one lingual (MPR grey scale), four buccal and one lingual (MPR inverted grey scale), and three buccal and four lingual (3D rendered).

### Validity

Table 1 presents bone thicknesses on the buccal and lingual surfaces of the root, as measured on photographs of the histological cuts, at the superior, visible position between the CEJ and the bone margin that had been recorded on the corresponding CBCT images.

**Table 1:** Bone thickness required (mm) to be visualized with various reconstruction techniques, viewing modes and resolutions compared to gold standard measurements

Reconstruction technique and viewing mode	Surface	Bone width (mm)		
		Standard^a^ Mean (SD)	High^b^ Mean (SD)	Gold standard Mean (SD)
CBCT, MPR grey scale	Buccal	0.219 (0.074)	0.173 (0.070)	0.094 (0.037)
	Lingual	0.563 (0.546)	0.356 (0.468)	0.097 (0.068)
CBCT, MPR inverted grey scale	Buccal	0.241 (0.101)	0.214 (0.068)	0.094 (0.037)
	Lingual	0.692 (0.658)	0.424 (0.454)	0.097 (0.068)
CBCT, 3D rendering	Buccal	0.202 (0.078)	0.183 (0.101)	0.094 (0.037)
	Lingual	1.139 (1.230)	1.208 (1.237)	0.097 (0.068)

CBCT, cone-beam computed tomography; CEJ, cementoenamel junction; MBL, marginal bone level; SD, standard deviation; MPR, multiplanar reformation; 3D, three-dimensional

^a^standard-resolution protocol; ^b^high-resolution protocol

Bone thickness measurements, based on reference measurements (CEJ-MBL) taken from high-resolution CBCT images viewed in MPR grey scale had the highest validity for both buccal (mean 0.173 mm) and lingual (mean 0.356 mm) surfaces when compared with the gold standard measurements (mean 0.094 and 0.097, respectively). Lowest validity, however, occurred for CBCT reference measurements in the standard-resolution, MPR, inverted grey scale viewing mode group for buccal surfaces (mean 0.241 mm) while for lingual surfaces, lowest validity occurred for reference measurements in the high-resolution, 3D rendering group (mean 1.208 mm).

**Fig. 3:**
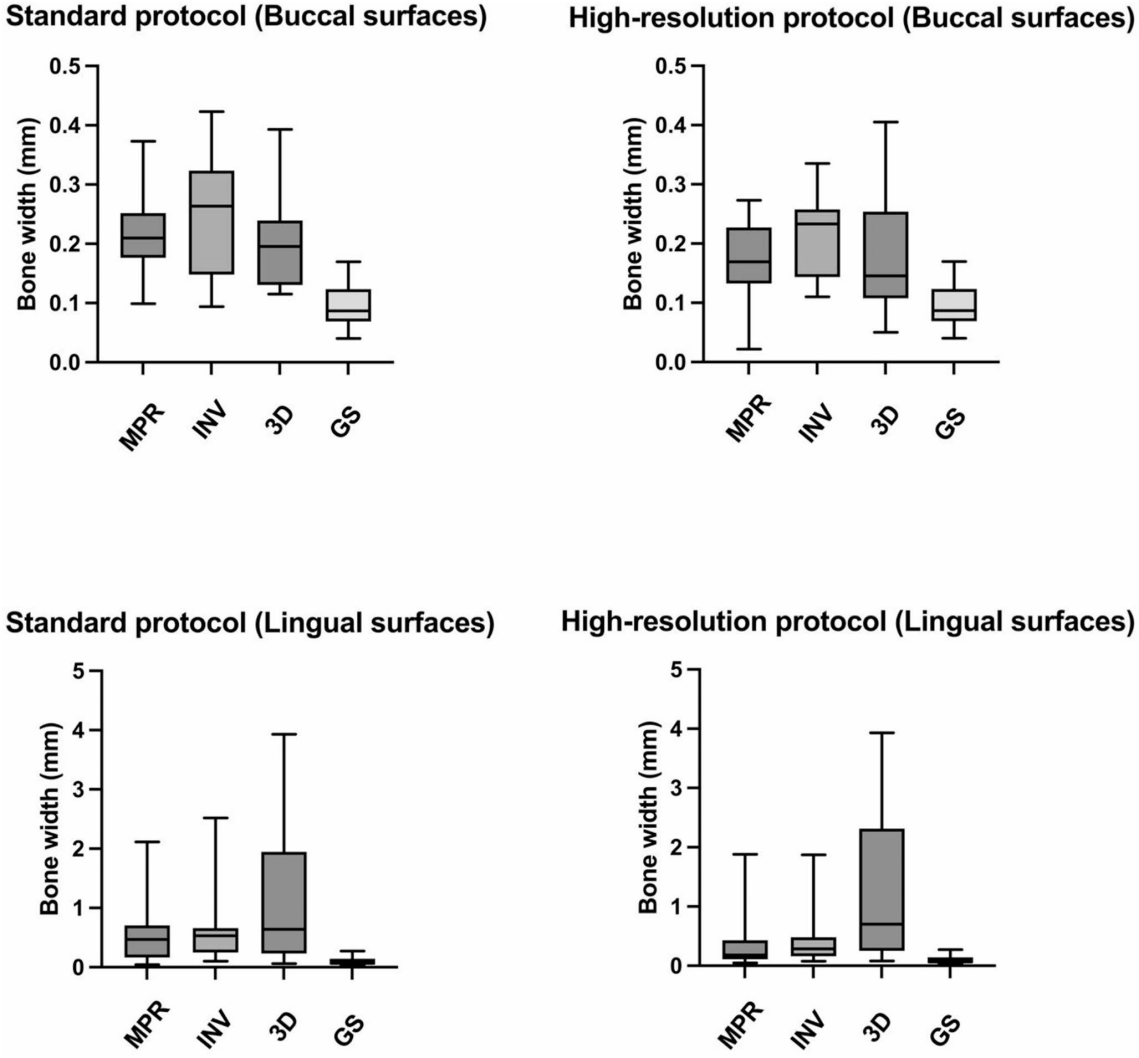
Boxplots presenting the median, maximum, and minimum measures of minimum bone thickness required in cone-beam computed tomography to be visible in images taken with a standard- or a high-resolution protocol (each protocol, *n* = 42), compared to gold standard measurements. *MPR*, multiplanar reformation in grey scale. *INV*, multiplanar reformation in inverted grey scale. *3D*, 3D-rendered. *GS*, gold standard. N.B. The y-axis representing the buccal surfaces increased by 0.1 mm compared to the y-axis of the lingual surface, which increased by 1.0 mm

The Fig. 3 boxplot illustrations of the data presented in Table 1 depict the likeness of the standard- and high-resolution data for the two surfaces in the three viewing groups. Included for comparison is a gold standard representation.

For buccal surfaces, mean bone thickness measurements obtained from the histological cut and based on CEJ-MBL reference measurements from the standard- and high-resolution MPR grey scale and 3D-rendered images agreed somewhat more closely with each other than when using reference measurements made on the MPR inverted grey scale images. For lingual surfaces, mean bone thickness measurements based on CEJ-MBL reference measurements from the standard- and high-resolution MPR images viewed in grey scale and in inverted grey scale agreed more closely with the gold standard than did the 3D-rendered CBCT images.

Mean lingual bone width using CEJ-MBL reference measurements from the 3D-rendered viewing group was measured as > 1.0 mm, regardless of resolution. Some surfaces in this group were observed to have a thickness of > 3.9 mm while the gold standards for these surfaces were observed to have no such thickness.

The difference between bone thickness required to be visualized and gold standard measurements for buccal surfaces using standard protocol are 0.108–0.147 mm, and 0.079–0.120 mm for high-resolution protocol. Differences between bone thickness required to be visualized and gold standard measurements for lingual surfaces using standard protocol are 0.466–1.042 mm, and 0.259–1.111 mm for high-resolution protocol, across the three viewing groups (MPR grey scale, MPR inverted grey scale, and 3D-rendering), respectively.

Significant differences were observed between the standard- and high-resolution protocols across all three viewing groups, compared to the gold standard, for both surfaces (Wilcoxon signed-rank test, *p* < 0.05).

## Discussion

This study evaluated buccal and lingual aspect bone widths at the distance from the CEJ where the marginal bone level was first visible, using different CBCT reconstruction techniques, viewing modes, and resolution protocols.

By comparing these measurements with a gold standard determined on histological photographs of a sliced tooth, we could study differences in accuracy among the various viewing-groups. Highest validity occurred with buccal aspect measurements in the high-resolution CBCT image group viewed in grey scale following MPR. Lowest validity occurred with lingual aspect measurements in the high-resolution, 3D-rendered CBCT image viewing-group.

Numerous studies on CBCT assessment of thin bone structures examine how image quality is affected when technical features, such as voxel size, are adjusted [11, 14]. In a controlled study, Cook et al. [15] compared accuracy between a long scan (619 projection images; 360-degree trajectory arch; 0.2 mm voxel size) and a short scan (169 projection images; 180-degree trajectory arc; 0.3 mm voxel size) to measure marginal bone dimensions. Because of the small difference in marginal bone measurements between the short and long scans, the short scan was deemed favorable due to the lower radiation dose. Molen [16] describes how the most effective way to combat partial volume averaging is to decrease voxel size, which in turn comes at the cost of requiring more radiation and the image being more prone to noise. The Molen study also addresses the difficulty of determining spatial resolution in comparisons using a phantom as the ideal. This is consistent with a study by Brüllmann et al., that the spatial resolution is reduced in a living person compared to a phantom [6]. The spatial resolution will always be less than the physical voxel size, and therefore is another technical feature affecting visualization of thin bone structures [6, 7, 17]. The present study utilized a voxel size of 0.125 mm which is optimized for the visualization of thin bony structures; thus, the results are valid under these specific conditions. However, different brands of CBCT systems utilize varying voxel sizes.

Studies on observer ability to detect marginal bone dimensions in CBCT images by modifying how bone is presented visually (by varying viewing mode, resolution, and reconstruction format) are scarce. One of the few studies on this issue, Lennholm et al. [12], sought to examine the effect of different resolution protocols (high- and standard-resolution), reconstruction techniques (MPR and 3D rendering) and viewing modes (grey scale and inverted grey scale) on the observer’s ability to detect the height of the MBL. Their study found that the viewing mode and reconstruction format with lowest validity was the 3D-rendered, high-resolution protocol for lingual surfaces, which agrees with the results of our study. However, in contrast to the present study, Lennholm et al. found highest validity for standard-resolution CBCT images viewed with MPR in inverted grey scale for buccal surfaces. Our study found those conditions to reflect buccal surface bone width with the lowest validity.

The 3D-rendered images in our study showed a greater variation in validity for lingual surfaces compared to the MPR grey scale and inverted grey scale viewing groups. These results are in line with both Lennholm et al. [12] and Fernandes et al. [18], who reported higher validity for MPR compared to 3D-rendered CBCT images. As CBCT does not use Hounsfield units, in contrast to conventional computed tomography (CT), the ability to distinguish between tissues of differing densities is more difficult. Therefore, the remaining amount of bone may alter because of varying screen settings and alteration. In the present study, the 3D-rendered buccal surfaces had similar (and in some cases, even better) validity than MPR grey scale images when compared to the gold standard. For both lingual and buccal surfaces, the difference in validity between MPR grey scale and MPR inverted grey scale images was low (Table 1).

In general, this study observed a tendency of CBCT imaging to overestimate the thickness of the superior marginal bone. Underestimation of the marginal bone height could explain this, as discussed in the Sun et al. [14] study on maxillary pig teeth. These researchers found an underestimation of bone height on CBCT images when bone width was less than, or close to, a voxel size of 0.4 mm. They also determined that a bone width of 0.6 mm was required to clearly distinguish the MBL. Our study used a voxel size of 0.125 mm and recorded a mean superior marginal bone width of 0.094–0.097 mm for our histological gold standard. This superior bone width below the voxel size might explain why most measurements from the CEJ to the MBL were overestimations, that is, alveolar bone height was underestimated.

In this study, radiological MBL was erroneously reported at a height where, on the histologic photographs, no marginal bone was visible. Partial volume averaging during CBCT image reconstruction might explain this overestimation of marginal bone height. This error occurred somewhat more frequently on the standard-resolution (21.4%) than on the high-resolution (19.0%) images. Partial volume averaging occurs predominantly at the border between structures of differing densities. As Molen [16] discussed, these structures are often smaller than the voxel size of the protocol. A voxel displays only one grey value at a time and is thus prone to partial volume averaging during the phase of CBCT image reconstruction. In our case, the relatively high density of the root surface may have overflowed to where the marginal bone level could be expected to be found, and was thus recorded as such. Differences in cut direction might also explain this error. If the direction of cut used for preparing the histological samples differs from the radiographic planes used during data reconstruction, readings of one image may not correspond to the histological, that is, the gold standard reading, with the result that the CBCT images depict bone that is not visible on the histological images.

Even though the high-resolution protocol had slightly greater accuracy in general compared to the standard-resolution images, the mean difference was low (Table 1). As the standard-resolution protocol delivers a lower radiation dose to the patient, it could be preferable when considering the As Low As Reasonably Achievable (ALARA) principle; the justified potential diagnostic benefit should be greater than the possible risk of the exposure [19]. This is in line with Lennholm et al. [12] and Ruetters et al. [20] who found differences between the two resolution protocols to be small in marginal bone measurements and of limited clinical consequence.

In the present study, it is observed that a bone thickness twice as great is necessary for visualization on the lingual side compared to the buccal side. This discrepancy may be attributed to the generally thinner nature of lingual bone in the anterior region of the mandible, which could result in a significant underestimation of bone presence in the lingual region, leading clinicians to erroneously conclude a lack of bone coverage. Conversely, this study also identified instances of bone overestimation, which may result in incorrect assumptions about bone coverage by clinicians. This knowledge is essential for the planning of orthodontic and prosthetic treatments, as it aids in the assessment of bone volume and avoidance of gingival reactions, both in the short and long term [21, 22].

A possible limitation of the present study is that each part is evaluated by a single observer. The inclusion of multiple observers could introduce inter-observer variability, potentially masking the true differences related to the method itself. However, a prior study investigating reliability in measurements of marginal bone height indicated that the observers demonstrated good agreement [12].

The samples used in this study were fixed during the CBCT scan, in contrast to the clinical situation where patient movement may cause motion or scatter artefacts due to greater variation in surrounding tissue and filling materials [23, 24]. Although this in vitro study is clearly an imperfect replication of the clinical situation, it represents a first-of-its-kind study in the field of research on perceived bone width and the effect of visualization parameters.

## Conclusion

We found that the best resolution protocol, reconstruction technique, and viewing mode for analyzing the buccal and lingual surfaces of the alveolar bone margin with CBCT are images exposed with a high-resolution protocol, reconstructed using MPR, and viewed in grey scale. These settings, valid for given conditions, were able to detect mean superior marginal bone widths of 0.173 mm at buccal surfaces and 0.356 mm at lingual surfaces– minimum bone widths needed for visibility on CBCT images. Twice the bone thickness is therefore required to be visualized lingually compared to buccally.

The accuracy of 3D-rendered images was higher at buccal than at lingual surfaces. However, use of 3D rendering is discouraged for measuring marginal bone since the risk of thin bone not being imaged increases. The accuracies of bone thickness estimations with MPR and using grey scale or inverted grey scale were similar, with the grey scale viewing mode being slightly more accurate.

## Data Availability

No datasets were generated or analysed during the current study.
